# The Impact of Pro-environmental Awareness Components on Green Consumption Behavior: The Moderation Effect of Consumer Perceived Cost, Policy Incentives, and Face Culture

**DOI:** 10.3389/fpsyg.2022.580823

**Published:** 2022-06-17

**Authors:** Minmin Shen, Jianhua Wang

**Affiliations:** School of Business, Jiangnan University, Wuxi, China

**Keywords:** pro-environmental awareness, green consumption, perceived cost, policy incentives, face culture

## Abstract

Based on the survey data of 839 consumers in Jiangsu and Anhui provinces, this article explores the formation mechanism and internal driving force of Chinese consumers’ green consumption, and clarifies the effect of consumers’ pro-environmental awareness components on green consumption and the moderating effect of perceived cost, policy incentives, and face culture. The results of the study show that pro-environmental awareness is the basis for green consumption. However, groups with pro-environmental awareness do not choose green consumption for sure. The transformation from awareness to behavior is also affected by many factors. Consumers’ perceived cost is an important obstacle to green consumption, while the face culture in Chinese society has a certain role in promoting green consumption. In this study, government policy incentives have no significant direct impact and moderating effect on consumers’ green consumption.

## Introduction

In recent years, resource constraints have been tightened, environmental pollution has become increasingly serious, and ecosystems have been continuously degraded. This not only constrains the development of social production, but also affects the quality of life for residents. Global environmental problems are mainly caused by human activities (Dong et al., 2017). The unrestrained consumption of various natural resources has brought a huge extra load to the earth, destroyed the ecological environment, and broke the natural balance (Maloney and Ward, 1973). The research of Mcdougall (1993) pointed out that unreasonable human consumption patterns have caused nearly 40% of the environment to deteriorate. Our individual actions have global repercussions, and our consumption is directly linked to the use of resources and the destruction of nature and its ecosystems (Mónica et al., 2020). Therefore, it is an inevitable trend and an important choice to build a green society and promote the greening of production and life in human society. In daily life, consumers changing traditional consumption patterns and practicing green consumption have an important positive effect on the construction and development of a green society.

Green consumption is a pro-environmental behavior, referring to that consumers pay attention to protecting the ecological environment in the three links of purchase, use, and disposal of commodities and weaken individual behaviors to have a negative impact on the environment as much as possible (Carlson and Kangun, 1993). Green consumption considers the needs of contemporary people and the needs of future generations. In order to promote green consumption, we need to sort out how consumer’s pro-environmental awareness affects green consumption and how the perceived costs of consumers and government policy interventions affect green consumption. In addition, everyone’s behavior is rooted in a specific cultural background, and consumer behavior is no exception (Wang, 2013). “Mianzi” is a concept with Chinese cultural characteristics (Zhao et al., 2018), and it is usually translated as “face concept” or “face culture.” Face culture is a cultural background variable that has the greatest impact on Chinese behavior (Wang, 2013), so it is also necessary to identify the effect of face culture on consumers’ green consumption behavior.

In this context, this study constructed an experimental model based on pro-environmental awareness, with perceived costs, policy incentives, and face culture as moderator variables, to explore the formation mechanism and internal driving force of Chinese consumers’ green consumption, and provide some theoretical bases and opinions for the development of China’s green market.

## Literature Review and Hypotheses

To study the impact of pro-environmental awareness on green consumption behavior, it is necessary to understand the psychological mechanism behind individual behavior. From the relevant literature, scholars have proposed different theories to explain individual behavior and the psychological mechanisms behind it.

The social psychologist Lewin (1976) proposed Lewin’s Behavior Model based on a large number of analytical experiments. He believes that the performance, tendency, and intensity of individual behavior are affected and restricted by both individual internal factors and external environmental factors. Individual internal factors are the specific conditions and characteristics inherent in an individual, including two basic dimensions: physiology and psychology. External environmental factors represent the external environment in which individuals live, including two basic dimensions: natural environment and social environment. Lewin’s (1976) Behavior Model states that human behavior is the product of the interaction between the individual and the environment. The model reveals and summarizes the general laws of human behavior to a certain extent, and has a high degree of generality and applicability. It has become the basic theory for the study of individual behavior.

Fishbein and Ajzen (1977) put forward the Theory of Reasoned Action (TRA) on the basis of the multi-attribute attitude theory, and in Ajzen and Fishbein (1980), further developed it. TRA argues that individuals are inherently rational, using all the information they can obtain in an orderly manner and taking action only after considering and weighing the meaning and consequences of taking a certain action. An individual’s behavior directly depends on the individual’s behavioral intention. Behavioral intention is the result of the combined effect of two factors, the individual’s attitude and subjective norm. Other factors can only indirectly influence an individual’s behavior through attitudes and subjective norms.

In order to explain and predict individual behavior more reasonably, Ajzen (1991) introduced the variable of Perceived Behavioral Control (PBC) on the basis of TRA and proposed the Theory of Planned Behavior (TPB). TPB measures how easy or controlled an individual is expected to feel when performing a particular action, similar to the concept of self-efficacy. When individuals perceive that they are more competent and have more resources, the more confident they will be to carry out their actions. TPB has been empirically tested by numerous studies, proving that the theory has high reliability and validity for analyzing individual behavior. However, TPB emphasizes the instrumental component of attitude, ignoring the emotional component of attitude, and to a certain extent reduces the theory’s explanation of individual behavior.

The Motivation–Opportunity–Ability Model (MOA) was proposed by Ölander and Thøgersen (1995), which explains the motivation and mechanism of individual behavior from three aspects: the subjective possibility (motivation), the objective possibility (opportunity), and the possibility of subjective individuals to know objective things (ability). The construction of MOA is based on two basic assumptions: one is to emphasize the important driving effect of motivation on individual behavior, and the other is to believe that the three elements of motivation, opportunity, and ability are complementary, and the three elements need to exist at the same time to achieve specific behavior. The individual’s motivation can directly affect behavior, and the two variables of ability and opportunity play a moderating role in the influence path from motivation to behavior. MOA is extremely inclusive and open, and it provides an effective analytical framework for later researchers to explore the dynamics of behavior.

Guagnano et al. (1995) found that the environmental-friendly behavior of individuals is the result of the interaction between environmental attitudes and specific situational factors when studying residents’ garbage recycling behavior. Based on this, a set of Attitude–Context–Behavior (ABC) theories was developed. Behavior is the interaction function of Attitude and Context. When the situational variables tend to be favorable, it may greatly promote the occurrence of residents’ environmental behavior; when the situational variables tend to be unfavorable, it may greatly hinder the production of environmental behaviors. The value of ABC is to demonstrate the relationship between the individual’s internal attitude elements and the external situational elements of the individual’s environment, and to verify that situational factors have moderating effects on the path of environmental attitudes to environmental behaviors.

To sum up, Lewin’s (1976) Behavior Model believes that behavior is the product of the interaction between the individual and the environment, and makes a basic induction and division of the individual’s internal elements and external environmental factors. TRA holds that individuals are inherently rational, using all the information available to them in an orderly manner, and take action after taking into account the meaning and consequences of taking a certain action. TPB, based on the theory of rational behavior, introduces the variable of PBC and believes that when individuals perceive their own abilities as stronger and possess more resources, they are more confident in carrying out their actions. In order to solve the problem that TPB does not consider emotional factors, based on the TPB, many scholars have introduced many variables, including emotional factors, into the model, and conducted continuous testing, supplementation, and integration, which greatly enhanced the model’s interpretation of individual pro-environmental behavior. MOA explains the motivation and mechanism of individual behavior from three aspects: the subjective possibility, the objective possibility, and the possibility of subjective to objective cognition. ABC holds that an individual’s environmental behavior is the result of the interaction between environmental attitudes and situational factors, and verifies that situational factors have a moderating effect on the path between attitudes and behaviors.

Based on the above model, Wang (2012) constructed an exploratory theoretical model, namely the Consciousness-Context-Behavior System Model, when he studied the low-carbon consumption behavior of Chinese consumers. The theory analyzes the dimensional structure and interaction effects of consciousness, situation, and behavior, and examines the moderating effect and direction of internal and external situational variables in the path of awareness to behavior. It is an extension and development of TRA and TPB, as well as a supplement to MOA and ABC. Empirical research pointed out that consumers’ low-carbon consumption awareness is an important pre-variable for low-carbon consumption behavior. The influence path of low-carbon consumption awareness to behavior is also moderated by internal context variables (individual implementation costs) and external context variables (social reference norms). In order to verify this model, Wang (2013) used the Consciousness-Context-Behavior System Model to explore residents’ resource-saving awareness and behaviors, and proved that this model is also valid in the research on residents’ resource-saving behaviors.

This study follows the Consciousness-Context-Behavior System Model, trying to construct a research framework to explore the green consumption behavior of Chinese consumers. In this model, consciousness is represented by environmental awareness (environmental knowledge, environmental affection, and environmental responsibility). Behavior is represented by green consumption. Context is the moderator variable in this model, represented by consumers’ perceived costs, policy incentives, and face culture.

### Role of Pro-environmental Awareness

Consumer behavior theory believes that individual consumer behavior is formed by a complex purchasing decision process under the influence of various stimulating factors. Green consumption, as an environmentally friendly lifestyle and consumption pattern, is also affected by many factors (Sheng et al., 2019). A large number of studies have proved that pro-environmental awareness has a significant role in promoting an individual’s green consumption (Wang, 2013; Liu et al., 2017; Eze, 2020). Environmental knowledge, environmental affection, and individual environmental responsibility are all important components of pro-environmental awareness (Liu et al., 2014; Mei et al., 2016; Vasiljevic-Shikaleska et al., 2018).

Ecological cognition is the driving factor of ecological consumption, and it can positively affect consumers’ ecological consumption (Fransson and Gärling, 1999). Having certain environmental knowledge is the prerequisite to generate environmental awareness (Maloney et al., 1975). Therefore, when consumers have a deeper understanding of the environment and environmental pollution issues, they are more inclined to adopt environmentally friendly green consumption. Research by Liobikien et al. (2016) proved that when consumers have a wealth of green consumption knowledge, they are more likely to adopt green consumption behaviors. Dursun et al. (2019) found that objective environmental knowledge’s effect can be used to break down young consumers’ psychological barriers that are unwilling to engage in pro-environmental actions, and to facilitate the change toward more sustainable energy consumption patterns.

The research of Han et al. (2007) pointed out that both cognition and affection can have effects on environmental protection behaviors, but the impact of affection on environmental protection is more direct and significant. Carrus et al. (2008) also reached a similar conclusion. Although environmental affection and environmental knowledge are both important components of pro-environmental awareness, environmental affection is more important than environmental knowledge. For example, in real life, some rural residents have insufficient environmental knowledge reserves due to low education, but they still bring positive affection to the environment. Newton et al. (2015) and Ritter et al. (2015) directly proved that environmental affection can significantly affect consumers’ willingness to purchase green products.

In addition, many studies have shown that consumers’ sense of environmental responsibility has a significant positive impact on the willingness to purchase green products. Environmental responsibility is the sense of responsibility and obligation that individuals have when they are willing to make efforts to solve ecological and environmental problems, and it is an important explanatory variable for pro-environmental behaviors (Sheng et al., 2019). Hines et al. (1987) proposed a model of responsible environmental behavior after a meta-analysis of 128 articles on pro-environmental behaviors. The research pointed that consumers with a high sense of environmental responsibility can better understand the relationship between themselves and the environment, and believe that it is everyone’s duty to solve environmental problems. Compared with consumers with low environmental responsibility, consumers with high environmental responsibility are more willing to adopt pro-environmental behaviors. A study on the consumer behavior toward energy-saving household appliances in China also proved that consumers’ environmental responsibility will drive them to pay attention to environmental issues and actively practice green consumption (Sheng et al., 2018). Therefore, this study proposes the following hypotheses:

*H1: Environmental knowledge has a positive impact on green consumption*.
*H2: Environmental affection has a positive impact on green consumption*.
*H3: Environmental responsibility has a positive impact on green consumption*.

The effect of one explanatory variable on the outcome variable will vary with the level of another explanatory variable (Wang, 2013). The effects of different components of awareness on behavior are not necessarily independent. The interaction effects of explanatory variables ultimately affect individual behavior choices. Tentatively, an explorative hypothesis is tested about the possible interaction effects between environmental knowledge, affection, and responsibility, in relation to green consumption.

*H4: Interaction effects between different components of pro-environmental awareness exist*.

### Role of Consumers’ Perceived Costs

However, pro-environmental awareness does not necessarily lead to pro-environmental behaviors. The conversion rate of eco-conscious to actual eco-consumption is far below the conversion rate between non-ecological awareness and behavior (Bamberg and Möser, 2007). Moderator variables can systematically change the form, strength, and direction of the relationship between the predictor and the criterion variable (Baron and Kenny, 1986; Aguinis et al., 2017). Thus, the study speculates that there may be moderator variables that influence the path from pro-environmentally awareness to behaviors. Several studies have demonstrated this speculation. Although ecological consumption can be achieved through intrinsic motivation efforts, it will also be limited by external conditions, such as household income, scale, and economic cost (Abrahamse and Steg, 2009). Zhang and Qing (2011) also found that energy consumption consciousness has an important influence on energy consumption in the study of Chinese residents’ energy consumption, but the degree of influence is interfered by the economy, households, and related groups.

The Motivation–Opportunity–Ability Model proves that individual motivation can directly act on behavior, while ability and opportunity have a moderating effect on the influence path from motivation to behavior. ABC verifies that contextual factors have moderating effects on the influence path of environmental attitudes to environmental behaviors. Wang (2013) conducted qualitative research on the resource conservation behavior of the Chinese public, and proved that internal and external context variables have a significant moderating effect on the influence path of conscious behavior. This study selects three contextual variables of consumers’ perceived cost, policy incentives, and social culture to specifically analyze the moderating mechanism of internal and external contextual variables on pro-environmentally aware green consumption behavior.

Consumers always make rational choices when it comes to environmental behavior. They always choose low-cost, high-yield options. Abrahamse and Steg (2011) classified garbage collection and support for an environmental policy as low-cost pro-environmental behaviors, while green consumption and the use of environmentally friendly equipment were classified as high-cost pro-environmental behaviors. Green products usually have a higher premium than ordinary products (Griskevicius et al., 2010). Also, green products are difficult to buy in traditional markets, and generally need to be purchased in supermarkets and large shops. This requires consumers to make certain sacrifices in terms of personal benefits, such as economy and convenience. Customer Perceived Value theory believes that when consumers make any purchase decision, they will weigh the total benefit and the total cost. Consumers also make the same value judgment when they conduct green consumption. The benefits of green products have an externality, and the value to the environment is higher than the value to the customers themselves. Green products with higher prices tend to weaken consumers’ concern and sense of responsibility for the environment, which in turn makes consumers with high price sensitivity reduce their willingness and behavior to purchase green consumption (Wang et al., 2015). In sum,

*H5: The relation between pro-environmental awareness and green consumption behavior will be weaker (stronger) for consumers with stronger (weaker) perceived costs*.

### Role of Policy Incentives

Policy incentives are important external situational factors that affect consumers’ green consumption. In order to protect the environment and promote the development of the green consumption market, the government would take a series of economic measures. For example, the government would reduce the production cost of green products through the reduction of taxes and stimulate consumers’ green purchase demand by preferential subsidies. But it is worth discussing whether the policy incentives are effective. Iyer and Kashyap ‘s (2007) research shows that the government’s incentive policies can effectively stimulate residents’ garbage collection. However, Slavin et al. (1981) and Abrahamse et al. (2005) pointed out that economic incentives can only temporarily stimulate energy-saving behaviors. Based on the literature, this study believes that policy incentives have a moderating effect on the conversion path of the consumer’s pro-environmental awareness to green consumption. In short,

*H6: The relation between pro-environmental awareness and green consumption behavior will be stronger (weaker) with stronger (weaker) policy incentives*.

### Role of Face Culture

When studying ecological consumption, the influence of cultural factors cannot be ignored (Liu et al., 2017). Culture is a comprehensive concept, covering every aspect of individual thinking and action, affecting people’s preferences and motivations, and influencing people’s behavior choices. According to this logic, pro-environment awarenesses and pro-environment behaviors are also rooted in a specific cultural environment. Face culture has a strong Chinese cultural flavor and occupies an important position in Chinese society. It is an informal system that describes the psychological process of interpersonal relationships in daily life (Wu, 2004). Huang (1985) believes that face culture refers to the social status or prestige that an individual has achieved because of his achievements in society. Face culture has a profound impact on Chinese consumption decisions (Pan et al., 2014). When the Chinese conduct consumption activities, there are some psychological motives for improving and maintaining self-image and preventing losing face (Zhu, 2006). The consumption of “mianzi” exists in all societies, including favor consumption, fashion consumption, conspicuous consumption, comparison consumption, and other types (Jiang, 2009). Therefore, we regard face culture as an important epitome of social culture and speculate that, under the effect of face culture, the public would pay attention to whether adopting green consumption can enhance their own personal image, and can demonstrate their personal qualities, economic conditions, and social status. In sum,

*H7: The relation between pro-environmental awareness and green consumption behavior will be stronger (weaker) for consumers with a stronger (weaker) tendency to face culture*.

**FIGURE 1:**
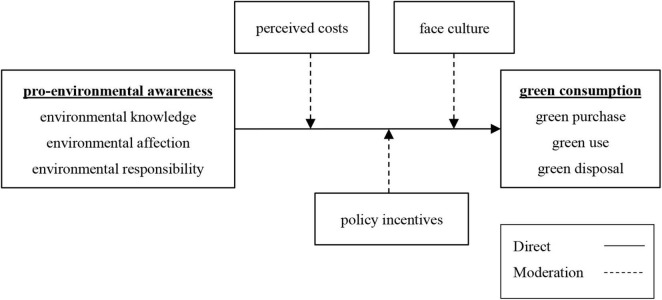
Hypothetical model of the study.

Regarding green consumption, most studies believe that green consumption focuses on reducing pollution, protecting the environment, and conserving resources and energy, including not only purchase behavior but also use and disposal behavior (Carlson and Kangun, 1993; Wang and He, 2011; Sun et al., 2015; Liu et al., 2017). Therefore, this study divides green consumption into three dimensions (green purchase, green use, and green disposal), investigates the direct impact of pro-environmental awareness (consisting of environmental knowledge, environmental affection, and environmental responsibility) on green consumption, and assesses whether the degree of its influence is moderated by consumers’ perceived costs, policy incentives, and face culture. The specific model assumption is shown in Figure 1.

## Research Design

### Measures

Environmental awareness includes three components, environmental knowledge, environmental affection, and environmental responsibility, and each one is measured by a specific scale.

The environmental knowledge scale mainly draws on the ecological cognition scale of Bohlen et al. (1993) and is measured using four items, including “I know oceans and rivers are being polluted,” “I know the hazards of the ozone hole,” “I know the pollution caused by pesticide residues to the soil,” and “I know the global warming is happening.”

The environmental affection scale mainly draws on the affection part of the ecological product selection scale of Fraj and Martinez (2007) and is measured using six items, including “I think most of the things I eat are contaminated with pesticides, which makes me very scared,” “Environmental pollution poses a great threat to the survival of animals and plants, which makes me very angry,” “The government has not taken more measures to control pollution, which makes me very indignant,” “The development of industry has caused serious pollution to the environment, which makes me very frustrated,” “I feel frustrated and angry about the smog,” and “The government has taken many measures to protect the ecological environment, which makes me very pleased.”

The environmental responsibility scale refers to the environmental scale of Fransson and Gärling (1999) and is measured using three items, including “Unless each of us recognizes the need to protect the environment, our future generations will bear the consequences,” “If each of us contributes a little to environmental protection, it will have a significant impact on the environment,” and “If everyone chooses green consumption, it is meaningful to environmental protection.”

The consumer perception cost scale mainly draws on the consumption perception risk scale of Jacoby and Kaplan (1972) and Mitchell and Greatorex (1993), measured using two items, including “I will worry that the value of the product is not worth its price” and “I will worry if this consumption is unsuccessful, it will waste my time to buy again.”

For the policy incentive scale, this study refers to the items in the research on ecological consumption awareness and behavior of rural residents by Liu et al. (2017), and is measured using two items, including “If there are discounts for buying eco-friendly products (low carbon, energy saving, etc.), I will choose to buy” and “If the government has subsidies, I would prefer to buy eco-friendly products (such as home appliances, solar energy, etc.).”

In terms of face culture, this study mainly refers to Pan et al. (2014) consumer value scale in the context of Chinese culture, and is measured using five items, including “I hope to show my best side in front of others so as not to be looked down on,” “Being rejected by others is a shameful thing,” “Do not directly or publicly accuse others of their mistakes,” “Even if the husband’s income is sufficient to support the family, the wife should also have her own career,” and “Appropriate praise can show respect for others.”

For the measurement of green consumption, the research has adjusted and amended the questionnaire based on the synthesis of relevant literature (Richins and Scott, 1992; Bohlen et al., 1993; Chan, 2001; Fraj and Martinez, 2007), combined with the context of Chinese culture and the actual situation, measured purchasing, using and disposing behavior in the process of green consumption with three items, including “I would recycle paper, glass, plastic bottles, cans, and other recyclable items,” “I would give priority to purchase environmentally friendly detergents, recycled paper products, etc.,” and “I have replaced a product I used before for environmental reasons.”

Each item is measured using the Likert scale and is assigned a value from 1 to 5 points, which correspond to the five levels of “strongly disagree” to “strongly agree” through individual subjective assignments.

### Sample Selection

Since a complete sampling frame was not available, a quota sampling method was used. In order to ensure the validity of the questionnaire, a small-scale pre-survey was conducted in the city of Wuxi before the formal investigation. The questions with unclear meaning and ambiguity in the questionnaire were adjusted and corrected based on the pre-survey feedback information. To ensure the authenticity of the investigation results, all investigators were uniformly trained before the formal investigation.

Data were collected in Jiangsu Province and Anhui Province, including four representative cities in Jiangsu Province (Southern Jiangsu: Wuxi; Central Jiangsu: Yangzhou; Northern Jiangsu: Huai’an and Lianyungang) and eight representative cities in Anhui Province (Southern Anhui: Xuancheng, Tongling, and Ma’anshan; Central Anhui: Anqing and Hefei; Northern Anhui: Huainan, Huaibei, and Fuyang). The sample selection range includes multiple prefecture-level cities with different geographic locations in the two provinces. In this sense, different places across geography were selected to ensure representative sampling.

In each surveyed city, the investigators identified respondents based on a 1:1:1:1 ratio of urban areas, suburbs, counties, and rural areas to ensure that the sample can cover consumers in all areas as much as possible. Then following the convenience sampling method, consumers aged 18 years old or above were selected to complete a face-to-face interview. Each interview took 2,030 min to finish. In total, 917 questionnaires were distributed. Among them, 839 of them were deemed valid, including 519 valid questionnaires from Jiangsu Province and 320 valid questionnaires from Anhui Province. The validity rate was 91.49%.

### Descriptive Statistics

The statistical data of the questionnaire show that women accounted for 55.30% and men accounted for 44.70% in terms of gender structure. The sample’s age distribution was relatively even, with the 18–35 age group accounting for 57.33% and 35 years old and above accounting for 42.67% of the sample. In terms of education distribution, junior high school and below accounted for 24.08%, high school or vocational school accounted for 25.39%, college or undergraduate accounted for 46.25%, and graduate and above accounted for 4.29% of the sample. In terms of family size, the sample was mainly based on the family size of 2–3 or 4–6 people, accounting for 35.28 and 58.88%, respectively. From the perspective of annual household income, the annual income of 66.03% of the sample was above 80,000 yuan, indicating that the majority of respondents have a relatively high standard of living. In terms of residence, urban areas accounted for 33.25%, suburbs accounted for 17.52%, counties accounted for 26.94%, and rural areas accounted for 22.29%. The sample residence distribution was relatively even.

### Reliability and Validity Test

The study used SPSS24.0 to analyze the internal reliability of the seven variables: environmental knowledge, environmental affection, environmental responsibility, perceived cost, government policy, face culture, and green consumption. The analysis results are summarized in Table 1. The Cronbach’s α coefficients of the variables are all above 0.7. It indicates the scale has comparable reliability to ensure good internal consistency between the variables. The pre-survey and modification of the questionnaire ensure the content validity of the questionnaire. In order to test the convergent validity of the scale, the researchers selected three test indicators: CR (construct reliability), KMO (Kaiser–Meyer–Olkin) value, and Bartlett’s test. The results show that the CR values of all items are above the standard value of 0.8, most of the KMO values are above the acceptable level of 0.7, and the significance level is 0.000, all of which pass Bartlett’s test. Among them, the KMO values of perceived cost and government policy are measured at 0.500, for the reason that these two items are composed of two observation variables. Since the other measurement indicators are perfect, this study believes that the scale validity of the scale passes the test.

**TABLE 1:** Reliability and validity test results.

Latent variables	Manifest variable	factor loading	CR	Cronbach’s α	KMO	Bartlett’s test		
						χ^2^	df	Sig
Environmental	EK1	0.869	0.914	0.874	0.815	1728.124	6	0.000
knowledge (EK)	EK2	0.895						
	EK3	0.809						
	EK4	0.837						
Environmental	EA1	0.593	0.843	0.773	0.815	1303.501	15	0.000
affection (EA)	EA2	0.612						
	EA3	0.825						
	EA4	0.815						
	EA5	0.700						
	EA6	0.557						
Environmental	ER1	0.788	0.847	0.728	0.676	523.730	3	0.000
responsibility (ER)	ER2	0.834						
	ER3	0.794						
Perceived cost (PC)	PC1	0.855	0.845	0.721	0.500	201.434	1	0.000
	PC2	0.855						
Government policy (GP)	GP1	0.929	0.926	0.841	0.500	629.035	1	0.000
	GP2	0.929						
Face culture (FC)	FC1	0.742	0.816	0.713	0.775	758.600	10	0.000
	FC2	0.717						
	FC3	0.524						
	FC4	0.791						
	FC5	0.639						
Green consumption (GC)	GC1	0.604	0.842	0.747	0.736	830.619	6	0.000
	GC2	0.758						
	GC3	0.852						

In addition, the study examines the discriminant validity between variables by comparing Pearson’s correlation coefficient between the variables and the AVE square root of each variable. The results are presented in Table 2. The absolute values of the correlation coefficients between the variables are smaller than the square root of the AVE of the variables listed on the diagonal. This means that the internal correlation between the observed variables is greater than the external correlation, and there is a high degree of discriminatory validity between the variables.

**TABLE 2:** Discriminant validity test results.

	Environmental knowledge	Environmental affection	Environmental responsibility	Perceived cost	Government policy	Face culture	Green consumption
Environmental knowledge	0.853						
Environmental affection	0.374**	0.879					
Environmental responsibility	0.592**	0.529**	0.853				
Perceived cost	−0.483**	−0.379**	−0.547**	0.794			
Government policy	0.509**	0.394**	0.635**	−0.489**	0.917		
Face culture	0.434**	0.331**	0.513**	−0.382**	0.459**	0.844	
Green consumption	0.441**	0.432**	0.488**	−0.576**	0.377**	0.445**	0.864

**Indicates significance at the level of 0.01, and the diagonal value is √AVE.

## Results

### The Main Effects and Interaction Effects of Pro-environmental Awareness Components on Green Consumption

In order to deeply explore the effect of pro-environmental awareness components on green consumption, the study used multiple regression analysis to test the main effect and interaction effect, and constructed the following multiple regression model:


$$\begin{array}{c} Z=\alpha +\alpha_{1}X_{1}+\alpha_{2}X_{2}+\alpha_{3}X_{3}+\alpha_{1i}X_{1}X_{2}+\alpha_{2i}X_{1}X_{3}\\ \;+\alpha_{3i}X_{2}X_{3}+\mu_{m} \end{array}$$

Among them, α is a constant term, α_*i*_ represents the regression coefficient, μ_*m*_ represents the error term, X_*j*_ represents the various components of pro-environmental awareness, and X_*j*_X_*k*_ represents the interaction of two components. Specifically, X_1_ represents environmental knowledge, X_2_ represents environmental affection, X_3_ represents environmental responsibility, X_1_X_2_ is the interaction between environmental knowledge and environmental affection, X_1_X_3_ is the interaction between environmental knowledge and environmental responsibility, and X_2_X_3_ is the interaction between environmental affection and environmental responsibility. The test results are presented in Table 3. Model I only considers the main effects of each component of consciousness on behavior, and model II analyzes the effects of pairwise interactions between various components of pro-environmental awareness on behavior in addition to the main effects.

**TABLE 3:** Test results of the main effects and interaction effects of various components of pro-environmental awareness on green consumption.

	Mode I			Model III		
	Standard regression coefficient	*T*-value	Sig.	Standard regression coefficient	*T*-value	Sig.
X_1_ (EK)	0.214	5.966	0.000	0.218	5.957	0.000
X_2_ (EA)	0.224	6.574	0.000	0.190	5.363	0.000
X_3_ (ER)	0.243	6.208	0.000	0.269	6.691	0.000
X_1_ × X_2_ (EK × EA)				0.004	0.107	0.915
X_1_ × X_3_ (EK × ER)				–0.014	-0.401	0.688
X_2_ × X_3_ (EA × ER)				0.099	2.730	0.006
R	0.556			0.564		
*R* ^2^	0.309			0.318		
Adj.*R*^2^	0.307			0.313		
F	124.722			64.577		
Sig.	0.000			0.000		

In the effect of pro-environmental awareness on green consumption, the impact of environmental knowledge, environmental affection, and environmental responsibility on green consumption is positively significant at the level of 0.001. Therefore, H1, H2, and H3 are confirmed. This shows that a certain amount of knowledge, a strong emotional expression, and individual responsibility for environmental issues effectively promote consumers’ green consumption in daily life.

From the perspective of interaction effects, the interaction between environmental affection and environmental responsibility is positively significant at the level of 0.01 for green consumption, while the interactions between environmental knowledge and environmental affection, and environmental knowledge and environmental responsibility are not significant. Therefore, H4 is proved. Interaction effects between different components of pro-environmental awareness exist. In this study, the interaction effect between environmental affection and environmental responsibility is confirmed.

**FIGURE 2:**
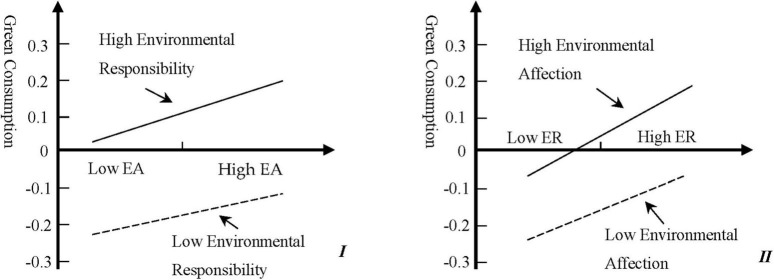
The interaction between environmental affection and environmental responsibility.

Based on the orthogonal interaction coefficients of environmental affection and environmental responsibility, we plotted Figure 2. It can be seen from Figure I that, for groups with a high degree of personal responsibility for environmental issues, enhancing their emotional perception of environmental issues can significantly increase the group’s preference for green consumption behaviors. From Figure II, we can see that groups with strong emotional responses to environmental issues, guiding them to correctly understand their relationship with the environment and cultivating environmental responsibility, can also play a positive role in inspiring their green consumption behavior.

### The Moderating Effect of Perceived Cost, Policy Incentives, and Face Culture

In order to examine the moderating effects of perceived costs, policy incentives, and face culture on the paths between pro-environment awareness components and green consumption behavior, this study selected hierarchical regression analysis and constructed the following multiple regression models:


$$\begin{array}{c} Z=\alpha +\alpha_{1}X_{1}+\alpha_{2}X_{2}+\alpha_{3}X_{3}+\alpha_{i}Y_{i}\\ \;+\alpha_{1i}X_{1}Y_{i}+\alpha_{2i}X_{2}Y_{i}+\alpha_{3i}X_{3}Y_{i}+\mu_{m} \end{array}$$

In the formula, α is a constant term, α_*i*_ represents the regression coefficient, μ_*m*_ represents the error term, X_*j*_ represents the various components of pro-environmental awareness, Y_*i*_ represents the moderator variable, and X_*j*_Y_*i*_ represents the moderating effect of the variable Y_*i*_ on the path relationship between X_*j*_ and Z, such as X_1_Y_1_ is the moderating effect of the perceived cost of consumers on the relationship between environmental knowledge and green consumption.

In model I, the moderating variables are not considered initially, and only the main effects of each dimension of awareness on behavior are analyzed. In model II, moderating variables are also included in the model and their main effects are analyzed. In model III, the study examines the moderating effects of perception cost, policy incentives, and face culture. The test results are presented in Table 4.

**TABLE 4:** Test results of the moderating effects of perceived cost, policy incentives, and face culture.

	Perceived cost			Policy incentives			Face culture		
	Mode I	Mode II	Model III	Mode I	Model II	Model III	Mode I	Model II	Model III
X_1_	0.214***	0.122***	0.109**	0.214***	0.205***	0.199***	0.214***	0.172***	0.187***
X_2_	0.224***	0.183***	0.165***	0.224***	0.221***	0.215***	0.224***	0.209***	0.184***
X_3_	0.243***	0.105**	0.118**	0.243***	0.221***	0.222***	0.243***	0.164***	0.170***
Y_*i*_		−0.391***	−0.388***		0.045	0.045		0.217***	0.227***
X_1_ × Y_*i*_			0.043			–0.001			–0.002
X_2_ × Y_*i*_			−0.092**			0.029			0.062*
X_3_ × Y_*i*_			0.023			–0.038			0.036
R	0.556	0.640	0.645	0.556	0.557	0.558	0.556	0.585	0.591
*R* ^2^	0.309	0.409	0.416	0.309	0.311	0.312	0.309	0.343	0.349
Adj.*R*^2^	0.307	0.406	0.411	0.307	0.307	0.306	0.307	0.340	0.344
F	124.722	144.382	84.479	124.722	93.923	53.751	124.722	71.803	63.738
Sig.	0.000	0.000	0.000	0.000	0.000	0.000	0.000	0.000	0.000

The symbols ***, **, and * indicates significance at the level of 0.001, 0.01, and 0.05, respectively.

Model I results are the same as summarized in Table 3. In model II, the main effect of perceived cost on green consumption is significantly negative at the level of 0.001. Consumers’ perceived cost has a significant inhibitory effect on green consumption behavior, for high premium and relatively few purchase channels. The main effect of face culture on green consumption behavior is significantly positive at the level of 0.001. This may be because, for some consumers, green consumption is a higher level of consumption, and green consumption can satisfy part of their vanity psychology. Moreover, today’s society advocates green consumption. When consumers who value “mianzi” more and think green consumption can win the “respect” of others, they will prefer green consumption.

**FIGURE 3:**
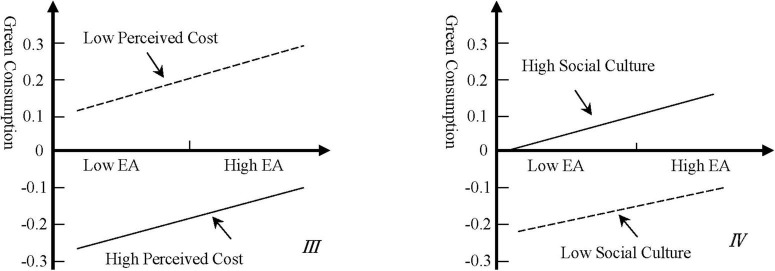
The moderating effect of perceived cost and social culture.

In model III, consumers’ perceived cost has a significant negative moderating effect on the path of environmental affection toward green consumption, while face culture has a weak regulating effect on the path of environmental affection toward green consumption. Therefore, H5 and H7 are confirmed. According to the moderating effect coefficient, we have drawn Figure 3.

It can be seen from Figure III that for groups with high perceived costs, the impact of environmental affection on green consumption is weaker than those with low perceived costs. The premium of green consumption and the inconvenience of purchasing are major obstacles for consumers to implement green consumption behavior. It can be seen from Figure IV that for groups with high face culture, enhancing their perception of environmental issues can effectively increase their probability of green consumption. Face culture has a certain role in promoting green consumption.

It is worth noting that the impact of policy incentives on green consumption is not significant, and they neither exert a main effect nor a moderating effect, which is contrary to our hypothesis. Therefore, H6 is rejected. It may be because the current green promotion policies are more oriented toward the production side and less incentive policies toward the consumer side, resulting in consumers not feeling significant about the policy incentives.

## Discussion

In order to explore the formation mechanism and internal driving force of Chinese consumers’ green consumption, clarify the impact of consumers’ pro-environmental awareness components on green consumption, and assess the moderating effects of consumers’ perceived cost, government policy incentives, and face culture, this article conducted a series of empirical analyses based on the survey data of 839 consumers in Jiangsu and Anhui provinces.

The results of the study show that pro-environmental awareness is the basis for green consumption. If the individual lacks pro-environmental awareness, it is difficult to adopt green consumption. Among the three components of pro-environmental awareness, that is, environmental knowledge, environmental affection, and environmental responsibility, all have a significant impact on green consumption behavior. The results are consistent with the research of Maloney et al. (1975); Hines et al. (1987), Fransson and Gärling (1999), Newton et al. (2015); Ritter et al. (2015), Liobikien et al. (2016), and Sheng et al. (2018). However, in Wang’s (2013) study of Chinese residents’ resource-saving awareness and resource-saving behavior, there is a significant positive interaction effect between knowledge and affection. In this study, the two components of environmental affection and environmental responsibility have positive interactions that promote each other. This shows that, although resource-saving behavior and green consumption behavior are both pro-environmental behaviors, there are certain differences in their influencing factors and paths.

In addition, groups with pro-environmental awareness are not necessarily choosing green consumption, and the transformation from awareness to behavior is also affected by many factors. Consumers’ perceived cost is an important deterrent to consumers’ green consumption. This is consistent with the findings of Wang et al. (2015). A consumer who supports green consumption may also be price sensitive. When there is an economic price to pay for green consumption, this willingness is significantly reduced. Due to the externality of the benefits of green consumption, when measuring the cost and value of green consumption, consumers pay more attention to their own efforts and ignore the benefits to the ecology, thereby reducing the willingness to green consumption. This is also in line with the behavioral characteristics of a “rational economic‘man.”

Cheng and Chen, 2020 research proves that, for Chinese rural residents, face culture plays a vital role in the transformation of non-environmental-friendly behaviors into environmental-friendly behaviors. This research also proves that face culture plays an important role in promoting green consumption for Chinese consumers. When consumers think that green consumption is a higher level of consumption, green consumption can satisfy their psychological motivation to gain respect and demonstrate their personal qualities, economic conditions, and social status.

However, in this study, the government’s policy incentives show that there is no significant direct impact and moderating effect on consumers’ green consumption behavior. This is inconsistent with the research results of Iyer and Kashyap (2007). We need to note that Iyer and Kashyap (2007) studied the residents’ garbage recycling behavior, while this article studies the residents’ green consumption behavior. Although garbage recycling behavior and green consumption behavior are both pro-environmental behaviors, there are still certain differences in the extent to which policy incentives affect them. It also may be due to the fact that current Chinese policies are more oriented toward green production, and the people on the consumption side do not have deep feelings about policy incentives.

These findings are important because they can guide us to better promote green consumption. In order to increase residents’ knowledge of environmental protection, strengthen their environmental concerns, and enhance their sense of environmental responsibility, it is necessary for the entire society to create a good atmosphere for environmental protection. In the long run, environmental protection education should be integrated into family education, pre-school education, primary education, secondary education, higher education, and other education systems. The knowledge of environmental protection should be popularized from an early age to establish the correct values of humans and nature. In the short term, the government can use various media channels (TV, radio, new media, community propaganda, etc.), with the help of publicity advertisements, environmental protection manuals, behavior guides, knowledge popularization, and other forms of communication, to discuss various environmental issues, strengthen public perception of environmental issues, and stimulate public environmental responsibility.

Considering consumers’ concerns about the high cost of green products, green product companies can weaken consumers’ attention to prices through product differentiation and brand positioning. Fundamentally, it is also necessary to accelerate scientific research and development for reducing the cost of green consumption.

Face culture is a cultural phenomenon unique to China. Taking advantage of this, the government can guide the public to give positive feedback on green consumption behavior. For example, the government can strongly praise and positively comment on green consumption behavior. Enterprises can focus on promoting the important role of green consumption in enhancing consumers’ personal image and quality of life, for encouraging the public to adopt green consumption practices.

## Conclusion

This research proves that pro-environmental awareness is an important basis for green consumption, and environmental knowledge, environmental affection, and environmental responsibility effectively promote consumers’ green consumption behavior. In particular, the study proves that there is a positive interaction between environmental affection and environmental responsibility. Environmental emotion and environmental responsibility act as an amplifier for each other. This is a contribution of this study. Because most of the existing literature tends to ignore the interaction of various components of pro-environmental awareness, to a certain extent, the conclusion of this study makes up for this defect.

This research also proves that perceived costs will hinder consumers from making green consumption, which is consistent with previous research results. However, this study also found that policy incentives have no significant impact on consumers’ green consumption behavior, which is inconsistent with previous research results. One of the reasons given in this article is that different types of pro-environmental behaviors have different influence factors and influence paths. This also enlightens us that, in future research, we can finely divide environmental behavior into different types, examining and comparing the influencing factors and paths of different types of pro-environmental behaviors.

Finally, the study proves that face culture plays an important role in the green consumption behavior of Chinese consumers. Under the long-term and profound cultural influence, the individual’s self-concept, cognition, affection, and motivation have been subtly affected. Most Chinese people will consciously identify and follow the Chinese cultural value system, norm system, and belief system. But face culture is only a part of Chinese culture, and only studying face culture has certain limitations. Future research can be extended to different characteristics of Chinese culture and study the effect of different Chinese cultural characteristics on the green consumption behavior of Chinese consumers.

## Data Availability Statement

The raw data supporting the conclusions of this article will be made available by the authors, without undue reservation.

## Ethics Statement

Ethical review and approval was not required for the study on human participants in accordance with the local legislation and institutional requirements. The patients/participants provided their written informed consent to participate in this study.

## Author Contributions

MS and JW contributed to the development of the theme and writing the manuscript. Both authors contributed to the article and approved the submitted version.

## Conflict of Interest

The authors declare that the research was conducted in the absence of any commercial or financial relationships that could be construed as a potential conflict of interest.

## Publisher’s Note

All claims expressed in this article are solely those of the authors and do not necessarily represent those of their affiliated organizations, or those of the publisher, the editors and the reviewers. Any product that may be evaluated in this article, or claim that may be made by its manufacturer, is not guaranteed or endorsed by the publisher.
